# An 8-month adapted motor activity program in a young CMT1A male patient

**DOI:** 10.3389/fphys.2024.1347319

**Published:** 2024-04-05

**Authors:** Giorgio Bottoni, Oscar Crisafulli, Caterina Pisegna, Marco Serra, Sara Brambilla, Fausto Feletti, Giovanni Cremonte, Giuseppe D’Antona

**Affiliations:** ^1^ CRIAMS Sport Medicine Centre Voghera, University of Pavia, Voghera, Italy; ^2^ Neurology Operative Unit, Civilian Hospital of Voghera, Voghera, Italy; ^3^ Department of Internal Medicine, University of Pavia, Pavia, Italy; ^4^ Department of Public Health Experimental and Forensic Medicine, University of Pavia, Voghera, Italy

**Keywords:** Charcot-Marie-Tooth, Neuropathy, Exercise, Balance, gait

## Abstract

**Background:**

It is unclear whether prolonged periods of training can be well tolerated. In Charcot-Marie Tooth disease (CMT). We report the effects of an 8-month, adapted motor activity (AMA) program in a 16-years-old CMT1A male patient. The program included strength, mobility, and balance training (two sessions per week, 1 h per session).

**Measures:**

Walking ability and walking velocity (Six-Minute Walking Test—6MWT, Ten Meters Walking Test—10 mW T), balance (Y-Balance Test—YBT, Berg Balance Scale—BBS), functional mobility (Short Physical Performance Battery—Short physical performance battery), fatigue (Checklist Individual strength questionnaire - CIS20R), health and quality of life (Short Form Health Survey 36 questionnaire—SF-36) were evaluated in three moments: before (T0), after 5 (T1) and 8 (T2) months of adapted motor activity. Dorsal and plantar foot flexion strength (Maximal Voluntary Contraction—maximum voluntary contraction) and neuromuscular functions (Electromyography—sEMG, interpolated twitch technique—ITT) were measured at T1 and T2.

**Results:**

Relative to T0, an amelioration of walking ability (6MWT, +9,3%) and balance (with improvements on Y-balance composite normalized mean reach of the right and left limb of 15,3% and 8,5%, respectively) was appreciable. Relative to T1, an increase in foot strength in three out of four movements (right plantar flexion, +39,3%, left plantar flexion, +22,7%, left dorsal flexion, 11,5%) was observed. Concerning voluntary muscle activation, a greater recruitment in the left, unlike right, medial gastrocnemius was observed.

**Conclusion:**

Results suggest the safety of an 8-month AMA program in a young patient affected by CMT1A.

## Introduction

Charcot-Marie-Tooth disease (CMT) is the most frequent hereditary neuropathy and one of the most common inherited diseases in humans, with an estimated prevalence of one in 2500 (McCorquodale et al., 2016). The term CMT refers to a group of hereditary, length dependent, motor and sensory polyneuropathies with a large genetic and phenotypic variability. The disorders are characterized by a progressive distal to proximal degeneration of peripheral nerves that determined weakness, atrophy, hypotonia and hypoesthesia. Particularly, in CMT1A, the most common form of CMT (Nam and Choi, 2019; Fridman and Saporta, 2021), the degenerative process is based on demyelination of axons, resulting in reduction of nerve conduction velocity (Nam and Choi, 2019; Fridman and Saporta, 2021). The phenotype can range from mild to severe functional limitations (Chetlin et al., 2004; McCorquodale et al., 2016), but most CMT patients have altered gait as well as balance problems, with frequent trips or falls (Anens et al., 2015). This, added to the greater energy demand (Menotti et al., 2011) could lead to sedentary lifestyle. A low level of physical activity in people with CMT and other neuromuscular disorders can increase the risk factors for comorbidities (Sman et al., 2015). Since there are no pharmacological therapies yet and although the optimal exercise modality and intensity remain still unclear, the CMT treatment involves physiotherapy and physical activity (Mori et al., 2019). In fact, exercise therapy for this population may slow the progression of symptoms (Mori et al., 2020) and may be beneficial to maintain strength and functional range of motion (Sman et al., 2015). Some evidence, suggest that strength (Chetlin et al., 2004), aerobic (Wallace et al., 2019), balance exercises or their combination appear possible intervention methods (Mori et al., 2019). However, to date it is unclear whether a prolonged combined exercise program can bring benefits without affecting the levels of fatigue and quality of life in CMT patients. Fatigue is a predominant component of CMT (Bellofatto et al., 2023) and has been reported as a key factor limiting physical activity in this population of patients (Anens et al., 2015). Although some data show that physical exercise can positively impact the fatigue levels of these patients (El Mhandi et al., 2008), a poorly dosed exercise program could increase the level of fatigue, possibly, triggering a vicious circle that would lead the patient to reduce daily physical activity with a consequent decrease in muscular efficiency and a negative impact on the quality of life (Roberts-Clarke et al., 2016). For these reasons, our aim was to study the effects of a prolonged AMA program based on combined training (strength, mobility and balance) in a sixteen-year-old male. The patient underwent a battery of walking, balance, strength, and neuromuscular functions tests before the beginning of the AMA program (T0), after 5 months (T1) and after 8 months (T2) of intervention. Our intent was also to study whether training can improve the level of voluntary activation by means of interpolated twitch technique (ITT).

## Case description

**FIGURE 1:**
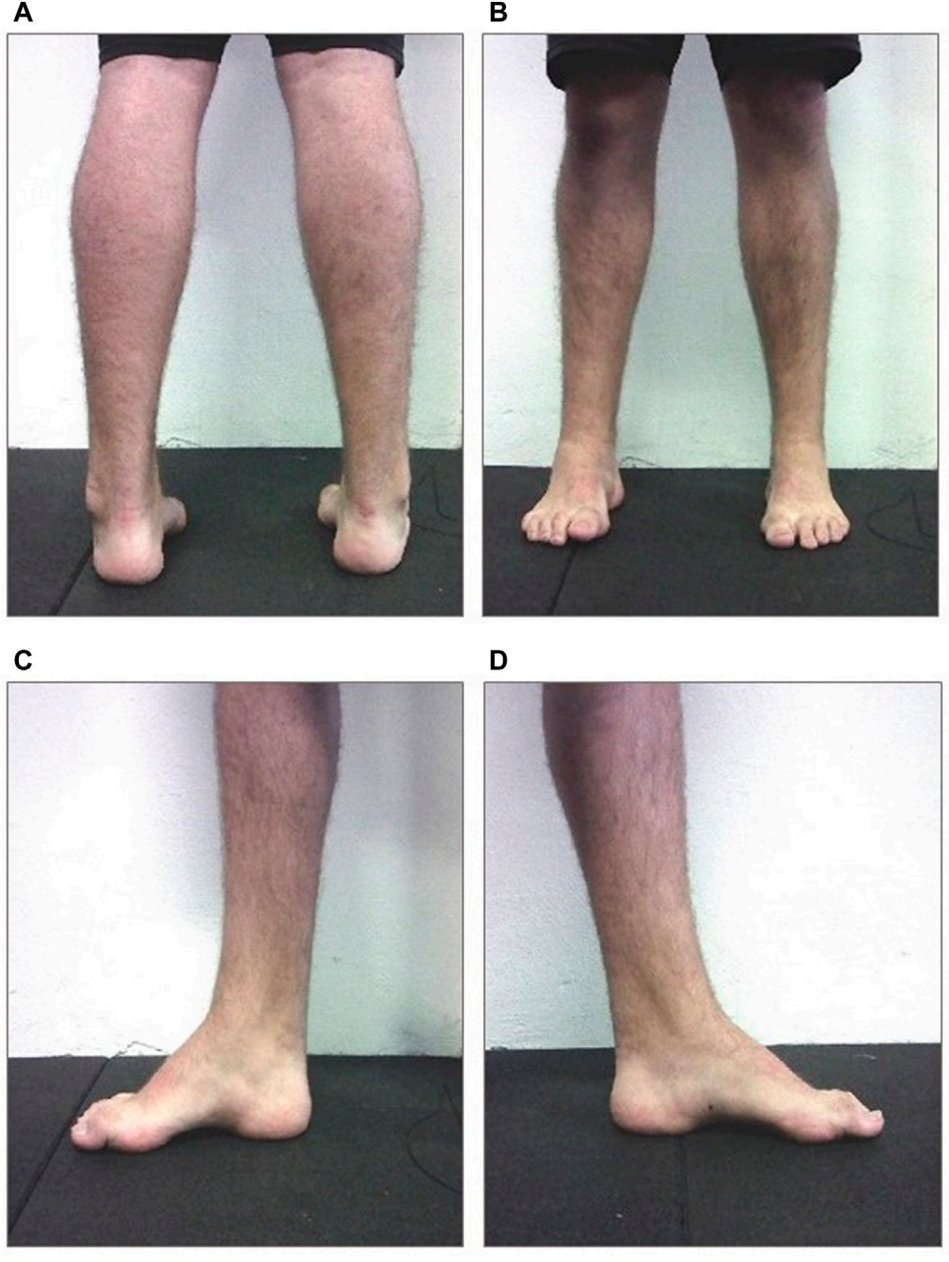
Image of the subject’s static plantar support. On the upper left panel **(A)** bilateral posterior view; On 440 the upper right panel **(B)** bilateral anterior view; On the lower left panel **(C)** medial view of the right foot; On 441 the lower right panel **(D)** medial view of the left foot (with permission from the subject).

The patient was a 16-years-old male, diagnosed with CMT1A, without comorbidities. The clinical suspect of the disease emerged at 1 year and half of life (2006) when the electroneuromyography (ENMG) reported the presence of a widespread slowing of motor and sensory conduction velocity with values lower than or equal to 50% of the average value compared to the normogram for age, with a slight reduction in compound muscle action potential (CMAP) and sensory action potential (SAP) in the lower limbs and lengthened distal motor latency. No signs of acute denervation were observed. A subsequent genetic analysis performed in 2010 highlighted the presence of heterozygous duplications of the PMP 22 gene exons one to five, confirming the previous clinical suspect. On 17 September 2021, he came to our centre for a medical-sports examination. He was 1.82 m, with a body weight of 80,3 Kg and a BMI of 24,2. Neurological examination outlined good motor function, with strength (segmental and global), trophism and muscle tone (proximal and distal) overall preserved both at the level of the shoulder and pelvic girdles. Normal and lively tendon reflexes in the upper limbs, not evocable at the patellar level (not even with facilitation manoeuvre) and symmetrically hypo evocable at Achilles tendons level. Deficit in dorsiflexion during standing were detected, especially on the right side. In the CMT neuropathy score (CMTNS) (Murphy et al., 2011) he obtained a total score of 3, one point in motor symptoms (legs) section and two points in strength (legs) section. The patient showed the classic CMT clinical presentation limited to the lower limbs, with hollow feet, hammer-like toes and varus heel (Figure 1). On 6th October, he began the AMA program, aimed at counteracting the progression of symptoms and improving the physical efficiency.

## Diagnostic assessment

Before the clinical and functional evaluations, the patient and his parents signed an informed content. The medical-sports examination performed prior to the beginning of the AMA program, which comprised also dynamic baropodometry, gait and mobility tests, highlighted.

• excessive right forefoot hyperload
• bilateral cavism, especially on the right.
• total absence of finger support with hammer-like toe marked on the right.
• evident reduced mobility in the right ankle joint.

Overall, the typical signs of CMT where more marked in the right side.

### Questionnaires and motor functions tests

At T0, T1 and T2 the patient underwent a battery of questionnaires dealing with the quality of life (Short Form Health Survey 36 questionnaire (SF-36)) (Apolone, 1997) and perceived fatigue (Checklist Individual strength questionnaire (CIS20R)) (Beurskens et al., 2000) and of functional tests regarding the walking ability (6 Minutes Walking Test (6MWT)) (ATS Committee on Proficiency Standards for Clinical Pulmonary Function Laboratories, 2002), functional mobility (Short physical performance battery (SPPB)) (Guralnik et al., 1994), and walking speed (10 m walk test (10 mW T) (Moore et al., 2018) and balance (Y balance test (YBT) and Berg balance test (BBS)) (Berg et al., 1992; Plisky et al., 2009; Shaffer et al., 2013). The values recorded at T0, T1 and T2 are summarized in Tables 1, 2.

**TABLE 1:** Absolute and percentage changes of the motor functions tests outcomes measured at the beginning, during and at the end of the AMA intervention.

Motor functions tests		T0	T1	T2	TO-T1 (%)	Tl-T2 (%)	**TO-T2** (%)
6MWT (m)		677	707	740	+4,4	**+**4,7	+93
10 mW T (m/s)	CS	1,28	1,44	1,33	+12,5	−7,6	+39
	FS	2.39	2.33	2.46	−2,5	+5,6	+29
YBT (%)	CNMRR	57,4	64	66,2	+11.5	+3,4	+153
	CNMRL	61.3	64.7	66,5	+5.6	+2.8	+8.5
BBS (score)		56/56	56/56	56/56	+0	+0	+0
SITI3 (score)		12/12	12/12	12/12	+0	+0	+0

6MWT, 6-min walking test, 6MWD, 6 minutes walking distance, 10 mW T, 10 m walking test; CS, comfortable speed; FS, fast speed (Moore et al., 2018), YBT, Y-balance test, CNMRR, composite normalized mean reach right leg; CNMRL, composite normalized mean reach left leg (Plisky et al., 2009), composite normalized mean reach was the sum of the three reach directions divided by three times limb length (Plisky et al., 2009), BBS.

**TABLE 2:** Absolute and percentage changes of the questionnaires scores measured at the beginning, during and at the end of the AMA intervention.

Questionnaires	Domains	T0	T1	T2	T0-T1 (%)	T1-T2 (%)	T0-T2 (%)
SF-36	PF (%)	100	100	100	+0	+0	+0
	PH (%)	100	100	100	+0	+0	+0
	EP (%)	100	66,7	66,7	−33,3	+0	−33,3
	EF (%)	45	55	60	+10	+5	+15
	EWB (%)	80	84	80	+4	−4	+0
	SoF (%)	100	100	100	+0	+0	+0
	P (%)	80	80	90	+0	+10	+10
	GH (%)	100	100	95	+0	−5	−5
	HC (%)	25	100	75	+75	−25	+50
CIS20R	Subjective Fatigue (score)	19/56	18/56	16/56	−1,8	−3,5	−5,3
	Concentration (score)	11/35	9/35	14/35	−5,7	+14,3	+8,6
	Motivation (score)	9/28	12/28	10/28	+10,8	−7,2	+3,6
	Activity (score)	4/21	5/21	5/21	+4,8	+0	+4,8
	Total (score)	43/140	44/140	44/140	+0,7	+0	+0,7

SF-36, Short Form Health Survey 36; PF, physical functioning; PH, role limitations due to physical health; EP, role limitations due to emotional problems; EF, energy/fatigue, EWB, emotional wellbeing, SoF, social functioning, P, pain, GH, general health; HC, health change, CIS20R = Checklist Individual StrengthTABLE, 3 Absolute and percentage changes of the lab test parameters measured during and at the end of the AMA, intervention.

### Muscular and neuromuscular functions evaluation

At T1 and T2, the patient underwent to tests evaluating the neuromuscular legs function. Participant lied in a supine position with the trunk slightly elevated and the foot firmly strapped to an ergometer (Tecnogym) maintaining the ankle joint at 90° (Solari et al., 2008). Bilateral ankle plantar and dorsi-flexion isometric contractions were performed. The following parameters were measured: maximum voluntary contraction (MVC) and time of endurance at 60% of the MVC (TOE).

Evaluation of the neuromuscular function was based on surface electromyography recordings (sEMG) and interpolated twitch technique (ITT) (Millet et al., 2011). Average Rectified Value (ARV) of the sEMG signals were obtained from gastrocnemius medialis and peroneus longus by means of bipolar electrodes (CDE-C 24 mm concentric, OT Bioelettronica, Torino). Before positioning, the skin, shaved if necessary, was gently abraded and cleaned with 75% alcohol to reduce electrical impedance. Electrode handling and positioning was in accordance to SENIAM guidelines (Hermens et al., 2000). ITT was used to determine the degree of nerve recruitment (PREVA). To this aim, a supramaximal electrically evoked single twitch was superimposed to a MVC (superimposed twitch) and compared to the potentiated response induced on the relaxed muscle by another supramaximal electrically evoked single twitch (control twitch) (Millet et al., 2011). Transcutaneous electrical stimuli were delivered to the tibial nerve using a high-voltage constant-current stimulator (DS7AH; Digitimer, Herthfordshire, United Kingdom). The degree of voluntary activation was obtained by using the linear equation: Voluntary activation (%) = [1—(superimposed twitch/control twitch)] × 100; (Shield and Zhou, 2004). Results are summarized in Table 3.

**TABLE 3:** Absolute and percentage changes of the lab test parameters measured during and at the end of the AMA intervention.

	Parameters		T1	T2	TI-T2 *(%*)
Right dorsiflexion	MVC (Kg)		12,8	12,2	−4,7
	TOE (s)		63,65	74,85	+17,6
Right plantar flexion	MVC (Kg)		20,7	28,9	+39,3
	TOE (s)		186,3	76,3	−59
	PREVA (%)		41,8	43,9	+2,1
	ARV $\left(1^{1/4}V\right)$	Pcroncus longus	144,8	18,1	−87,5
		Medial gastrocncmius	10,6	185,8	+1646,2
Left dorsiflexion	MVC (Kg)		11,72	13,07	+11,5
	TOE 0)		115,4	39,6	−65,5
I ell plantar Ilexion	MVC (Kg)		29,44	36,12	+22,7
	TOE (s)		226,6	113	−50,1
	PREVA (%)		62,2	83,0	+20,8
	ARV $\left(1^{1/4}V\right)$	Peroncus longus	96,1	174,3	+81,4
		Medial gastrocncmius	12,4	62,8	+406,4

MVC, maximum voluntary contraction; TOE, time of endurance; PREVA, voluntary activation before the fatigue task; ARV, average rectified value; Î¼V, microvolt.

## Adapted motor activity program

Based on the functional evaluation, which highlighted a lack of strength in the lower limbs and difficulties in maintaining balance and walking, a two sessions week of 1 h/session AMA program was structured with a combination of strength, mobility, and balance training with the specific aim of counteracting such impairments. The training consisted in three subsequent phases. *First phase* (October 2021–November 2021): the first part of the session consisted of a 10 min (min) warmup bike routine for ankle mobility and low-intensity aerobic exercise. Mobility and stretching training was 15 min of ankle and hip mobility exercises, static and dynamic stretching of the gastrocnemius, soleus, gluteus, hip flexors, quadriceps, hamstrings, and tensor fasciae latae. Balance and proprioception training consisted of 10 min of monopodalic work with support on the ground or on a trampoline, and bipodalic work on unstable surfaces (Skimmy, Navaris), and walk with an alternate gait pattern, with complete roll of the foot, heel–toe walking and walking on the forefoot. Strength training consisted of 25 min of exercises performed with low intensity isometric or concentric contractions with medium–high repetitions: bridge, planks, squat (3 sets of 40 s (s)) bipodalic calf (3 sets of 20 s), total body resistance exercise (TRX) traction, and reinforcement of knee and ankle flexor and extensor muscles with elastic bands, especially the peroneal muscles and tibialis anterior (3 sets of 12–15 repetitions (rep)). At the end of this phase, the patient gained articular range of motion of the ankle and good functionality of knee and ankle.

*Second phase* (December 2021- February 2022). The first part of the session consisted of a 10 min warm-up bike routine for ankle mobility and medium-intensity aerobic exercise. Mobility and stretching training was 15 min of ankle and hip mobility exercises, static and dynamic stretching of the gastrocnemius, soleus, gluteus, hip flexors, quadriceps, hamstrings, and tensor fasciae latae. Balance and proprioception training consisted of complex tasks such as maintaining monopodal balance on the ground while reaching for an object placed on the ground or touch objects positioned around him with his free limb, and bipodal work on unstable surfaces (Skimmy, Navaris) with the addiction of complex tasks (eyes closed or bouncing a ball against the wall). Strength training consisted of 25 min of interval training, usually split into two circuits of four exercise. The exercises were carried out at medium repetitions (three sets of 8–12 rep) chosen from: double support squat, split squat, bulgarian split squat, hip trust, deadlift, bipodalic and monopodalic calf with overloads, push up, planks, total body resistance exercise (TRX) traction, dumbbell exercises to strengthen the chest, shoulders, biceps and triceps, and reinforcement of leg muscles with stronger elastic bands than in the previous phase. *Third phase* (March 2022-May 2022). The first part of the sessions consisted of 10 min of moderate intensity aerobic exercise on cyclette. Mobility and stretching training was 15 min of ankle and hip mobility exercises, static and dynamic stretching of the gastrocnemius, soleus, gluteus, hip flexors, quadriceps, hamstrings, and tensor fasciae latae. Balance and proprioception training consisted of complex tasks such as maintaining balance after jump on the ground or on trampoline and monopodalic work on unstable surfaces (Skimmy, Navaris). Strength training was 25 min of interval training, usually split into two circuits of four exercise. The same exercises as in the previous phase were performed, but with increasing load and lower repetitions (three/four sets of 6–10 rep).

## Discussion

To the best of our knowledge, this is the first report in which an 8-month AMA program is carried out in a CMT1A patient. After training on a twice-weekly basis, significant improvements in balance, gait and leg muscles strength were observed, without negative sequels on fatigue perception and quality of life.

Data on YBT would support a substantial balance improvement. In fact, compared to T0, the CNMR of both right and left limb increased at T2 by 15,3% and 8,5%, respectively. The greater improvement in the right lower limb (the most affected by the disease) allowed to reduce the initial difference in balance between the two limbs (see table 1). These results suggest that balance training may be useful in counteracting the loss of balance in CMT1A patients, in line with some previous works (Kobesova et al., 2012; Liu et al., 2018). Data on 6MWT would indicate an improvement of walking capacity both at T1 and T2 which resulted, at the end of the treatment, in a 9,3% increase in walking distance, compared to baseline. The comfortable speed and fast speed values of the 10 mW T underwent an alternation of improvements and worsening (see table 1). However, the T0-T2 comparation show an amelioration of 3,9% and 2,9%, respectively. Although some specific exercises for walking were carried out only in the first phase of the intervention, seems likely that the observed gait improvements are still attributable to the exercise program, as it is known that the outcomes regarding balance and walking ability are strongly correlated in the patients (Mori et al., 2019).

Most domains of the SF-36 did not change significantly during the intervention period. However, the score of “energy/fatigue” domain increased both at T1 and T2. Further, the “health change” domain score increased at T1 and, despite a slight decrease between T1 and T2, at the end of the intervention remained higher than the values at T0. In this light, it seems plausible that the exercise therapy program had a positive impact on the subject’s perception of fatigue and on his health status; this would be in line with previous results obtained in various patient’s cohorts (Nightingale et al., 2018; Tous-Espelosín et al., 2020). The total score of CIS20R did not change significantly during the intervention period. However, the score of “subjective fatigue” domain decrease both at T1 and T2. This evidence seems to confirm the results obtained in the “energy/fatigue” domain of the SF-36, supporting the possibility of a positive impact of exercise therapy on the subject’s perception of fatigue. The muscular and neuromuscular evaluation showed meaningful variations in several parameters. The MVC improved in the right plantar flexion (+39,3%), left plantar flexion (+22,7%) and left dorsiflexion (+11,5%) while slightly decreased in the right dorsiflexion (−4,7%). The increase of MVC in three out of four movements could be due to the peculiar adaptation to resistance training, in which muscle fiber hypertrophy, with consequent strength augmentation, is known to occur (Lopez et al., 2021; Carvalho et al., 2022). Conversely, the TOE decreased in the right plantar flexion (−59%), left plantar flexion (−50,1%) and left dorsiflexion (−65,5%) while slightly increased in the right dorsiflexion (+17,6%). These results suggest an inverse relationship between MVC and TOE that seems to reflect the principle according to which an increase in expressed force is associated with a decrease in the contraction maintenance time (West et al., 1995; Pagliano et al., 2018). On the contrary, the slight reduction in MVC and the consequent increase in TOE observed in right dorsiflexion could be attributable to the pathological course. This seems consistent with the clinical evaluation which immediately highlighted a major involvement of the right lower limb. The EMGs results show substantial changes in the recruitment’s degree of muscles involved in plantar flexion. Indeed, at T1, in both limbs, the peroneus longus was the more active muscle, possibly trying to compensate for the low level of activity of the medial gastrocnemius. On the contrary, at T2 there was an important augmentation in the level of activation of the medial gastrocnemius, so much so that it becomes the predominant muscle in the right leg (see table 3). The PREVA of the right tibial nerve remained substantially unchanged (+2,1%) while significantly improved in left tibial nerve (+20,8%). The cross-referencing of these data with those relating to EMGs, MVC, balance and gait suggests that physical activity, despite having no effects on the primary cause of the pathology (i.e., nerve degeneration), could lead to improvements in terms of strength and function. Specifically, these data would indicate that the improvement in left plantar flexion strength could be due to both improved nerve recruitment and fiber hypertrophy of the tested muscles (Andersen and Aagaard, 2000; for a review see Folland and Williams, 2007) while the improvement in right plantar flexion strength could be only due to muscle fibers hypertrophy. Noteworthy, the post-AMA improvements were observed in the less clinically compromised body areas. Considering that the pathological course foresees that, over time, the areas involved by the disease may increase (Shy et al., 2005), this would suggest that the early phases of the disease may be the most suitable for engaging in AMA in order to obtain more significant results.

### Limitations

Our data, reported for a single case, cannot be generalized to other patients with CMT, especially considering the heterogeneous clinical presentation of the disease and do not allow us to accurately quantify how much the reported improvements derive from the proposed intervention. Moreover, due to technical constraints, we were not able to perform an EMG evaluation at T0. Therefore, although the reported data would still provide indications about the patient’s adaptation to the proposed AMA program, these refers to a shorter time frame than the entire intervention. Certainly, future studies on a large sample of clinically homogeneous patients compared to a control group will allow us to measure the difference in the rate of amelioration between healthy and diseased subjects.

## Conclusion

Although the nature of the study does not allow us to determine the effectiveness of the proposed AMA program, the lack of negative sequelae regarding fatigue and quality of life suggests its safety. The positive data observed in the post-AMA evaluations, while not relatable to the proposed intervention, encourage the conduction of further studies regarding the effects of AMA in wider cohorts of CMT patients.

## Data Availability

The raw data supporting the conclusion of this article will be made available by the authors, without undue reservation.
